# Lightweight Image Restoration Network for Strong Noise Removal in Nuclear Radiation Scenes

**DOI:** 10.3390/s21051810

**Published:** 2021-03-05

**Authors:** Xin Sun, Hongwei Luo, Guihua Liu, Chunmei Chen, Feng Xu

**Affiliations:** 1 School of Information Engineering, Southwest University of Science and Technology, Mianyang 621010, China; sunxin@mails.swust.edu.cn (X.S.); ccm@swust.edu.cn (C.C.); xufeng@swust.edu.cn (F.X.); 2 Shenzhen Launch Digital Technology Co., Ltd., Shenzhen 518000, China; lhw@launchdigital.net

**Keywords:** nuclear radiation scenes, lightweight image denoising, texture retention, attention, receptive field block, Mish, asymmetric convolution

## Abstract

In order to remove the strong noise with complex shapes and high density in nuclear radiation scenes, a lightweight network composed of a Noise Learning Unit (NLU) and Texture Learning Unit (TLU) was designed. The NLU is bilinearly composed of a Multi-scale Kernel Module (MKM) and a Residual Module (RM), which learn non-local information and high-level features, respectively. Both the MKM and RM have receptive field blocks and attention blocks to enlarge receptive fields and enhance features. The TLU is at the bottom of the NLU and learns textures through an independent loss. The entire network adopts a Mish activation function and asymmetric convolutions to improve the overall performance. Compared with 12 denoising methods on our nuclear radiation dataset, the proposed method has the fewest model parameters, the highest quantitative metrics, and the best perceptual satisfaction, indicating its high denoising efficiency and rich texture retention.

## 1. Introduction

In an environment with intense ionizing radiation, energetic particles can easily damage the electronic and optical components of image sensors [1], causing the captured digital images to be highly degraded; the images are essential for the subsequent professional analysis or other advanced computer vision tasks, e.g., image classification [2], object detection [3], and semantic segmentation [4], etc. Although shielding measures such as covering sensors with lead boxes [5] can improve the radiation resistance level to a certain extent, these measures will increase volumes and workloads of the perception machines sharply or raise the costs of these alternative sensors. Therefore, it is wise to remove strong noise quickly and retain textures as much as possible for the captured radiation scene images by micro-chips, i.e., to focus on effective and robust denoising algorithms.

Few researchers pay attention to denoising algorithms in terms of radiation scene images with complex shapes and dense distributions. Wang et al. [6] proposed an improved median filtering method combining adaptive thresholds and wavelet transformations to effectively reduce the nuclear radiation noise. Zhang et al. [7] used adaptive segmentation and fast median filtering to denoise nuclear radiation noises. Yang et al. [8] combined the frame difference method with interpolation algorithms to restore nuclear radiation images. These real-time denoising methods focus on nuclear radiation noise removal, but they find it difficult to handle very strong noises caused by extreme high radiation dosage.

Traditionally, a captured degraded digital image *Y* can be modeled as *Y* = *Y*′ + *N* [9,10]. Assuming that the degradation factor is additive noise *N*, a clean image *Y*′ can be restored through *Y*′ = *Y* − *N*. We carefully analyzed nuclear radiation images and found that the noise in the images is also additive. Therefore, in addition to the above existing denoising methods for radiation scenes, general denoising algorithms focused on additive noise removal may also have good denoising performances in radiation scene images. The existing general image denoising methods fall into two categories: image prior-based methods [11,12,13,14,15] and discriminative learning methods [16,17,18,19,20,21].

Image prior-based methods use some prior information of natural images for noise removal, e.g., local smoothness, non-local self-similarity, sparsity, etc. A Block Matching and 3D filtering (BM3D) algorithm [11] utilized the non-local self-similarity prior to natural images, and it was one of the state-of-the-art (SOTA) image prior-based methods. In a different way, Dong et al. used the sparsity prior to natural images and proposed the Nonlocally Centralized Sparse Representation (NCSR) algorithm [12] for noise removal. Gu et al. proposed a denoising algorithm named Weighted Nuclear Norm Minimization (WNNM) [15], combining the Non-local Means method (NLM) [13] with Low-rank Representation (LRR) [14]. Though theoretically clear, these SOTA image prior-based methods are time consuming due to their multiple iterations. Last but not least, the verbose hyperparameters of these methods, e.g., the size of sliding windows, the number of image blocks, and the supposed noise variances, vary from one denoising scene to another. Therefore, general image prior-based methods are not suitable for the denoising task in radiation scenes which require high denoising efficiency.

Discriminative learning methods use hard constraints between noised-and-clean image pairs to remove the complex noises without specific mathematical definitions. A landmark of discriminative learning methods was Denoising Convolutional Neural Networks (DnCNN) [16], which applied Convolutional Neural Networks (CNN), residual learning and batch normalization techniques to remove Additive White Gaussian Noise (AWGN) for the first time. On the basis of DnCNN, Fast and Flexible Denoising Network (FFDNet) [17] adopted the learned noise level map as a part of the network input to improve the denoising effect. After that, the Convolutional Blind Denoising Network (CBDNet) [18] used 5-layer Fully Convolutional Networks (FCN) to adaptively obtain the noise level map, which was hugely different from FFDNet and greatly enhanced the blind denoising ability. Recently, the Batch Renormalization Denoising Network (BRDNet) [19] adopted dilated convolutions and batch renormalizations to achieve a balance between training efficiency and model complexity. In addition, the Attention-guided Denoising Network (ADNet) [20] applied an attention block at the end of a lightweight backbone and obtained the best denoising results. Benefiting from convolutional feature extractors, Graphics Processing Unit (GPU) computing, and end-to-end training, the CNN-based denoising methods had better performance than traditional image prior-based methods in terms of efficiency and usability, so they are suitable for nuclear radiation scenes with complex noises.

However, the fast CNN-based denoising networks find it difficult to achieve a balance between model complexity and the denoising effect. Moreover, these CNN-based methods pay little attention to texture retention, so the results are prone to being smooth. To solve the texture problem, Details Retraining CNN (DRCNN) [21] added a texture learning unit on the basis of DnCNN and its promising qualitative results proved that the retraining strategy was useful. Nevertheless, the network structure and optimization method of DRCNN was so simple that its noise learning ability was limited. In short, there is still a lack of the image restoration network with high denoising efficiency and good texture retention.

Taking the rapidity and information retention into account with respect to the strong nuclear radiation noise removal task, we design a lightweight CNN-based denoising network composed of a Noise Learning Unit (NLU) and a Texture Learning Unit (TLU). The NLU adopts the effective non-local idea from traditional image prior-based methods and uses the popular residual learning technique from CNN-based methods in a novel way. To be more specific, the backbone of NLU bilinearly consists of a Multi-scale Kernel Module (MKM) and a Residual Module (RM), obtaining non-local information and high-level texture information, respectively. Moreover, both the MKM and RM have few channels, and their bottoms have Receptive Field Blocks (RFB) and Attention Blocks (AB) to expand receptive fields and enhance convolutional features. In addition, a small sub-network named the Texture Learning Unit (TLU) is at the end of the NLU. It uses an independent loss for optimization and learns detailed texture features simply and effectively. The entire network uses the Mish activation function to obtain good nonlinearity. At the same time, asymmetric convolutions are applied throughout the whole network to greatly reduce the amount of model parameters. The main contributions of our work are as follows.

1. We designed an extreme lightweight denoising network that not only effectively and efficiently removes the complex and strong nuclear radiation noises, but also carefully retain its texture details.
2. We applied useful tricks from other computer vision tasks like multi-scale kernel convolution, receptive field blocks, Mish activation and asymmetric convolution to image denoising for the first time. Detailed experiments proved that these techniques benefit image restorations.
3. The network has good generalization and performs well in other denoising tasks. Compared with the six popular CNN-based denoising methods in removing synthetic Gaussian noises, text noises, and impulse noises, the proposed method still has the highest quantitative metrics.

The rest of this paper is organized as follows. Section 2 analyzes the nuclear radiation noise. Section 3 introduces the methodology, including the overall network framework, detailed structures of the sub-networks, and the adopted deep learning tricks. Section 4 introduces the experiments and Section 5 analyzes the results. Section 6 performs detailed discussions and Section 7 draws the conclusion.

## 2. Analysis of Nuclear Radiation Noises

We analyzed the noise of the nuclear radiation scene images to guide our research method. The studied noised images were captured by special robots in a real nuclear emergency accident. Note that these images are all polluted by nuclear radiation noises, and there is no original clean image without pollution.

**Figure 1:**
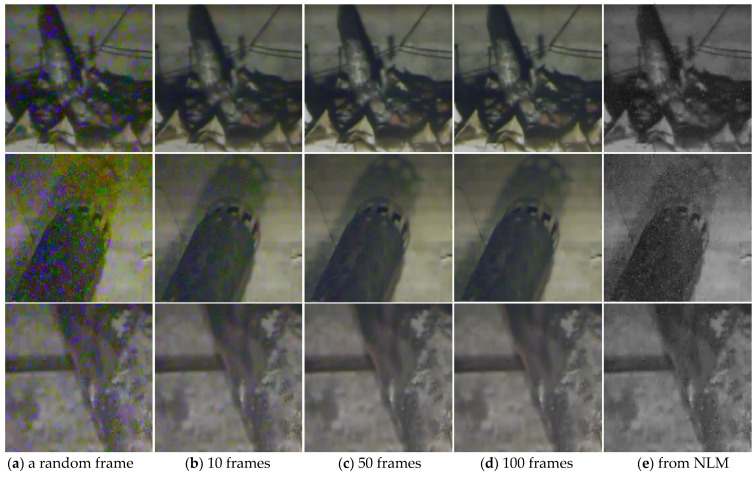
The averaged results and the denoised results. (**a**) Shows the original noised images randomly selected in frame sets. (**b**–**d**) Show the results of averaging 10, 50, 100 frames, respectively. (**e**) Shows the denoising results from the Non-local Means method (NLM).

The analysis idea comes from the studies of real noise removal [9,10,18], that the clean ground truth image can be obtained by averaging noised photographs with the same lens. Figure 1 shows the averaged results of multiple frames and the denoised results from the NLM [13] of three challenging nuclear radiation scenes.

It can be seen from Figure 1a that the nuclear radiation noises have irregular shapes and distributions, which is quite challenging. As shown in Figure 1e, the NLM which works well for AWGN hardly removes nuclear radiation noises, indicating that the noises should not be simply defined as a kind of Gaussian noise or impulse noise, and traditional image prior-based methods find it difficult to handle the denoising task. It is worth noticing that, in Figure 1b–d, averaging frames does have obvious denoising effects on the nuclear radiation scene images, and with the increase in the averaging numbers, the mean images are prone to being cleaner. The intuitive results demonstrate that the nuclear radiation noise has the same properties as additive noises which means the averaging operation can greatly reduce the noise variances [9].

**Figure 2:**
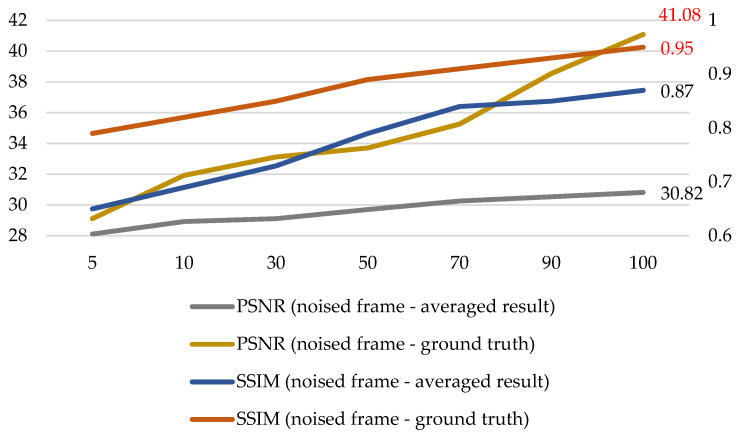
Qualitative relationship between the averaging number and the quantitative metrics.

In order to find more solid evidence that the nuclear radiation noise is additive, we analyzed the qualitative relationship between the averaging number and the quantitative metrics, as shown in Figure 2. The ground truth images were obtained by averaging 150 frames, and the metrics are peak signal-to-noise ratio (PSNR) and structural similarity (SSIM).

It can be seen from Figure 2 that with the increase in the averaging number, PSNR and SSIM metrics between the randomly selected noised frame and the averaged result are prone to being higher; with the increase in the averaging number, the averaged result becomes closer to the clean ground truth. Meanwhile, it can be seen in Figure 2 that, when the averaging number is 100, the PSNR value reaches 41.08 and the SSIM value reaches 0.95. The three cues indicate that the nuclear radiation noise is almost addictive, and the clean ground truth images obtained by averaging 150 frames are reliable. Therefore, we can make a nuclear radiation dataset composed of noise-and-clean image pairs and handle the difficult denoising task with general CNN-based methods.

## 3. Methodology

Existing general denoising methods such as those in [11,16] do have good performances in additive noise removal, but they are not effective enough in denoising radiation scene images due to the complex noise shapes and high noise density. In this case, we design a lightweight CNN with robust noise learning ability and sufficient texture retention. The proposed method can also be regarded as a general image restoration method as it has good performances in removing synthetic Gaussian noise, text noise, and impulse noise. The implementation details are as follows.

### 3.1. Overall Network Framework

The network consists of a noise learning unit (NLU) and a texture learning unit (TLU). The NLU consists of two parallel sub-networks with different feature extraction structures, i.e., the multi-scale kernel module (MKM) and the residual module (RM). To enlarge the receptive field and enhance the abstract features, the bottoms of the MKM and RM are connected with receptive field blocks (RFB) and spatial attention blocks (AB) in sequence.

**Figure 3:**
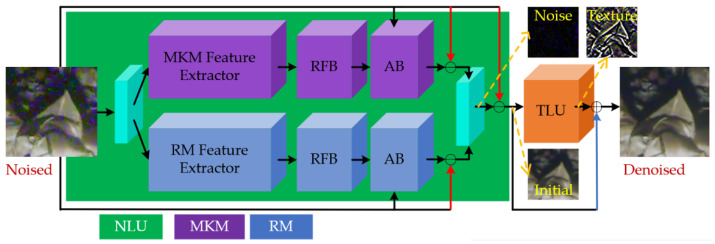
The overall network framework. The purple pipeline represents the multi-scale kernel module (MKM), the dark blue pipeline represents the residual module (RM); the green area is the first sub-network noise learning unit (NLU), and the orange area is the second sub-network texture learning unit (TLU). The light blue parts represent the input or output convolution layers, playing the role of feature fusing. The red arrows indicate element-wise subtractions, and the blue arrow indicates an element-wise addition.

In order to describe the network pipeline concisely, mathematical descriptions of the key stages are given. The input of the NLU is noisy images marked as *Y*, and the output of the NLU is the initial denoised map without texture retention marked as *Y*_1_. Meanwhile, the input of the TLU is *Y*_1_, and its output is the ultimate denoised image with rich texture details marked as *Y*_2_. RFB are defined as the function *f_rfb_*, AB are defined as *f_ab_*, and the TLU is defined as the function *F_TLU_*. The complete mathematical description of the whole forward network is as follows.

(1) $$\left\{\begin{array}{l} Y_{1_{M}}=Y-f_{ab}(f_{rfb}(Y,f_{1}(Y)))\\ Y_{1_{R}}=Y-f_{ab}(f_{rfb}(Y,f_{2}(Y)))\\ Y_{1}=Y-f_{3}(Y_{1_{M}},Y_{1_{R}})\\ Y_{2}=Y_{1}+F_{TLU}(Y_{1}) \end{array}\right.$$

where *Y*_1*M*_ and *Y*_1*R*_ denote the outputs of the MKM and RM, respectively; *f*_1_ and *f*_2_ denote their corresponding feature extractors, and *f*_3_ denotes a feature fusing block implemented by a concatenation and a 3 × 3 convolution. Figure 3 shows the framework in a more intuitive way.

As shown in Figure 3 and Equation (1), the skip connections in the NLU are all subtractions, as the NLU learns residual noise by *N* = *Y*_1_ − *Y*, while the skip connection in the TLU is addition, as the TLU obtains the ultimate denoised image by *Y*_2_ = *Y*_1_ + *T*, where *N* and *T* denote the latent noise and textures, respectively. Though addition and subtraction operations can both fit the network in theory, our skip connection strategies make the network easy to train as they are based on the mathematical meaning of the addictive noise and addictive textures. The whole network is end-to-end training and fully convolutional; that is, the ultimate denoised image *Y*_2_ is directly obtained from the original noised image *Y* without any intermediate operations and conversions. The following subsections will describe the above parts in further detail.

### 3.2. Noise Learning Unit

#### 3.2.1. Feature Extractors

MKM Feature Extractor. The MKM feature extractor in the NLU is to learn multi-scale non-local information of the image. Convolutions are similar to the spatial filters in some traditional image prior-based methods, e.g., the NLM [13], so CNN can also latently learn self-similarity features of natural images to remove noises. In order to enhance the denoising effect, we adopt multi-scale convolution kernels just the same as traditional multi-scale filters. Specifically, the feature extraction part of MKM is composed of 4 Inception-like structures [22] connected in series, and each Inception-like structure is parallelly composed of 3 convolutions with different kernel sizes. We adopt the same data preprocessing strategy as other CNN-based denoising methods do: feeding small image patches sized 50 × 50 into the network rather than the full image so as to augment the training set and make full use of GPU in the training stage. Furthermore, the sizes of the convolution kernels we implemented are 1 × 1, 3 × 3 and 5 × 5, which would avoid the receptive field overflowing and make the calculation parameters moderate.

RM Feature Extractor. The RM feature extractor in the NLU is to learn high-level texture information of the image, and this part is composed of 4 small residual blocks. The residual blocks are similar to the Bottlenecks in [23]. First, the input map of a residual block follows a 1 × 1 convolution to reduce the channels. Next, abstract features are obtained by a 3 × 3 convolution and an activation function. Then, a 1 × 1 convolution is used to increase the channels to a specified dimension. Finally, a subtractive skip connection is performed to connect the input and the output of this block. The skip connections can solve the problems of gradient disappearance and latently learn the residual noise, so as to improve the training efficiency.

In order to further expand the receptive field, a dilated 3 × 3 convolution is added between each Inception-like block or residual block in the MKM and RM modules. It is worth noting that we did not expand the receptive field through verbose dilated convolutions (e.g., 14 dilated convolutions in series in [19]) but added just one dilated convolution between the blocks. The reasons are as follows:

1. The superposition of multiple convolution kernels with the same dilation rate will cause some pixels not to participate in feature extraction all the time, which is unfriendly for the pixel level prediction task, i.e., image denoising.
2. We fully consider the parameters of the network and use few dilated convolution layers to make the network lightweight. To ensure that the final receptive field is still large, other cheap tricks are added in the MKM and RM.

**Figure 4:**
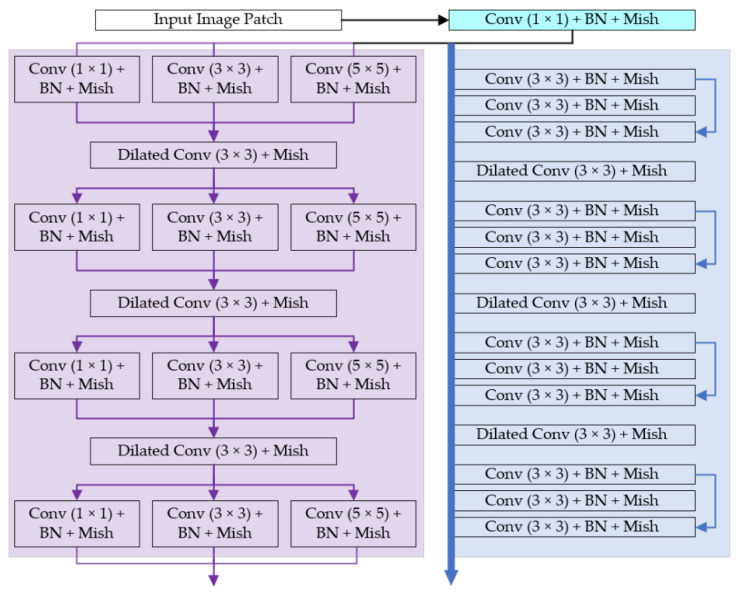
The structure of the MKM feature extractor and the RM feature extractor. The input is a batch of noised image patches sized 50 × 50. The purple part belongs to the MKM, containing 4 Inception-like blocks; the blue part belongs to the RM, containing 4 residual blocks. The activation method of each convolutional layer is Mish, and the filter number of each layer is 32. Except the dilated convolution layers, all convolutional layers are followed by batch normalizations.

Figure 4 shows the details of the feature extraction parts in the MKM and RM. Note that we implemented a novel activation method named Mish rather than the popular Rectified Linear Unit (ReLU). In addition, some convolutional kerels are decomposed to reduce the parameters of the model, i.e., we adopt the asymmetric convolution strategy. The padding methods are all the “SAME”, and the channel numbers are all 32 (rather than 64, generally implemented in other denoising methods [16,17,18,19,20,21]). The Mish activation and asymmetric convolution will be introduced in the following subsections.

#### 3.2.2. Feature Enhancements

In order to obtain a larger receptive field and learn more abstract features in a cheap way, we added lightweight receptive field blocks (RFB) and spatial attention blocks (AB) behind the feature extractors.

Receptive Field Block. The RFB is originated from the RFBNet [24], proposed for the task of real-time object detection. The main idea of the RFB is to simulate the mechanism of human vision through taking the dilated rates and the eccentricities of dilated convolutions into consideration, so as to cheaply enlarge the receptive field with a small parameter increase. Some recent works [9,19,20] concluded that the receptive field is essential for image denoising, so receptive field blocks are added at the bottom of our feature extractors.

**Figure 5:**
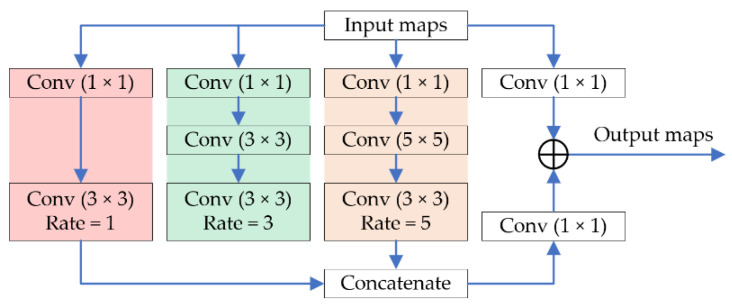
The Receptive Field Blocks (RFB) module. The inputs are the feature maps obtained by the MKM feature extractor or the RM feature extractor. The output is the result of an element-wise addition between the input maps followed by a 1 × 1 convolution and the concatenation from three branches. Note that the channel number of our RFB module is 32.

As shown in Figure 5, the RFB is also an Inception-like structure, i.e., paralleling several convolution layers with multi-scale kernels. Different from the traditional Inception blocks, RFB adopts dilated convolutions with multi-scale kernels, and the corresponding dilation rates are carefully designed. Moreover, a short cut is performed in RFB, and the feature fusing method is also different from vanilla Inception blocks, i.e., fusing Inception-like features through a concatenation rather than an element-wise addition. Experimental results show that the RFB improves our denoising effect, while it keeps our model lightweight. In addition, the number of basic channels in the RFB we set is 32 rather than 64 in RFBNet, so as to reduce the parameters of our model.

**Figure 6:**
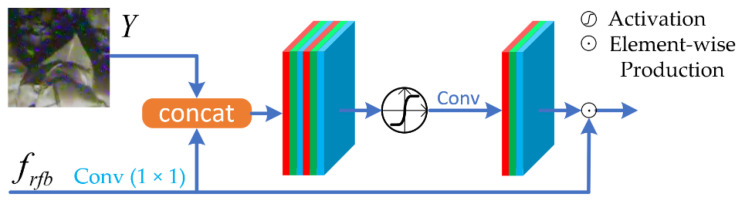
The attention blocks (AB) module. The inputs are the original image patches and the feature maps from the RFB, then they are concatenated with the help of a 1 × 1 convolution. The concatenated maps are followed by a 3 × 3 convolution and a Tanh activation. After feature fusing, an element-wise production is performed between the feature maps from the RFB branch and the Tanh branch, as the output of this attention block.

Attention Block. We implemented attention blocks (AB) at the end of RFBs, so as to further enhance the learned features and enlarge receptive fields. AB make the network pay more attention on the noised regions, and selectively enhances the feature map. In computer vision tasks, the attention mechanism is generally implemented in two ways: Spatial Attention (SA) [25] and Channel Attention (CA) [26]. First, SA and CA receptively score the feature map with activation functions in the spatial domain and channel domain. Then, the enhanced feature maps could be obtained by element-wise productions between the original feature maps and the score maps. We noticed that global or average pooling operations in CA would lose plenty of image information, which is harmful for the image restoration task, though this kind of dimensional reduction strategy is useful in other computer version tasks [27]. Therefore, we use the SA mechanism instead of the CA to better improve the denoising performance. The inputs of our attention block are feature maps from an RFB and the original image patches. The mathematical description of AB is as follows.

(2) $$f_{ab}=Y\times f_{act}(f_{cat}(Y,f_{rfb}))$$

where *Y* is a batch of noised image patches, and *f_rfb_* is the result of an RFB followed by a 1 × 1 convolution. *f_cat_* denotes a concatenation followed by a 3 × 3 convolution, and *f_act_* denotes the activation function Tanh with the value range of (−1, 1). The attention block uses element-wise productions to achieve weight assignments, so their training and inference stages can also be accelerated by a GPU or Neural-network Processing Unit (NPU). Note that our activation function is Tanh instead of Sigmoid, as we take some negative information into consideration. Figure 6 shows the AB in an intuitive way.

### 3.3. Texture Learning Unit

The popular CNN-based denoising networks, e.g., DnCNN [16], FFDNet [17], BRDNet [19], ADNet [20], etc., all learn the latent noises through subtractive skip connections, rather than directly learning the mappings between noised images and clean images as other image restoration tasks do, e.g., Single Image Super Resolution (SISR) [28]. It is undeniable that the denoising methods adopting residual learning are easy to train and have high quantitative metrics. However, these methods inevitably lose rich texture details and may have unsatisfactory perceptual results. In this case, DRCNN [21] finds that the denoised image with rich texture details *Y*_2_ can be modeled as *Y*_2_ = *Y*_1_ + *T*, where *Y*_1_ and *T* are the unsatisfactory initial denoised map and the learned texture details, respectively. We borrow this idea and add an NLU with an addictive skip connection at the end of the TLU, but we only used 3 convolution layers for texture learning instead of the 17 layers performed in DRCNN. The reason is that our loss functions in the NLU and TLU are different. The loss in the NLU has already obtained rich feature information from back propagation, so it is unnecessary for the NLU to extract high-level texture features through a heavy subnetwork. The reliability of this approach has been proven in knowledge distillation tasks [29]; that is, the loss values can make up for the gap between large models and small models in terms of feature representation abilities. In this way, our network economically learns rich texture details.

In denoising tasks, the choices of loss functions vary from scene to scene. Previous works [10,30] have deduced that the expectations of the popular loss functions *l*_0_, *l*_1_, and *l*_2_ are the mode, the median and the mean value, respectively. Hence that *l*_0_ loss is suitable for impulse noise removal in Magnetic Resonance Imaging (MRI) images [31,32], and *l*_1_ loss is suitable for removing the text noises such as the watermarks in photographs. It is worth noticing that *l*_2_ loss measures the distance of two pixels more accurately than other losses, so it is beneficial for some mapping tasks such as SISR and our texture learning unit (TLU). As for our NLU, the choice of loss functions is up to the noise type, e.g., gaussian noise, text noise, impulse noise, and our nuclear radiation noise. In short, the whole network uses a joint loss to optimize in the training stage:

(3) $$L_{j}=\lambda_{1}\times L_{n}+\lambda_{2}\times L_{t}$$

where *L_j_* is the joint loss, *L_n_* is the loss for the NLU, and *L_t_* is the loss for the TLU; *λ*_1_ and *λ*_2_ are two weighting coefficients for *L_n_* and *L_t_*, respectively.

### 3.4. Other Tricks

Mish activation. Activation functions introduce nonlinear components into neural networks, greatly enhancing the feature learning abilities. The most commonly used activation function is the Rectified Linear Unit (ReLU), which alleviates the gradient vanishing problem through a simple function, i.e., *x* = *max*(*x*, 0). However, in image restoration networks, the operation that directly assigned the negative values to 0 will lose plenty of mapping information [10]. Although some methods [24,33] used Leaky ReLU to retain some negative information, the nonlinearity of these methods became weaker than the vanilla ReLU, which is not beneficial for noise learning. Therefore, we use a novel activation function named Mish [34], which retains some negative information, and maintains the excellent performance of nonlinearity as ReLU. The mathematical description of Mish is as follows.

(4) $$f_{\mathrm{mish}}=x\cdot \tanh(\ln(1+e^{x}))$$

Asymmetric convolution. Although the number of parameters of our network is less than some of the popular methods due to the lightweight feature extractor, we need to further reduce the parameters so as to denoise the nuclear radiation scene images faster in embedded platforms. In this case, we used asymmetric convolutions [35] to further simplify the model. Specifically, we replaced all 3 × 3 convolutions with serial combinations of 3 × 1 and 1 × 3 convolutions and replaced all 5 × 5 convolutions with serial combinations of 5 × 1 and 1 × 5 convolutions. This strategy significantly reduced the parameters and improved the inference speed.

In addition, we implemented batch normalizations behind all convolution layers except several dilated convolutions. Previous works [16,19] have shown that batch normalizations make the data distributions more regular, which help the network fit and improve the denoising effects. Except for the two input layers in the NLU and TLU, all convolution layers have no bias. The purpose is to reduce the addition times and improve the inference speed.

## 4. Experiments

In this section, we first introduce the experimental datasets, including our nuclear radiation dataset and two popular public datasets for synthetic noise removal. Then training and testing details of our experiments are listed. Finally, we introduce the evaluation strategies, including objective evaluation and subjective evaluation.

### 4.1. Nuclear Radiation Dataset

**Figure 7:**
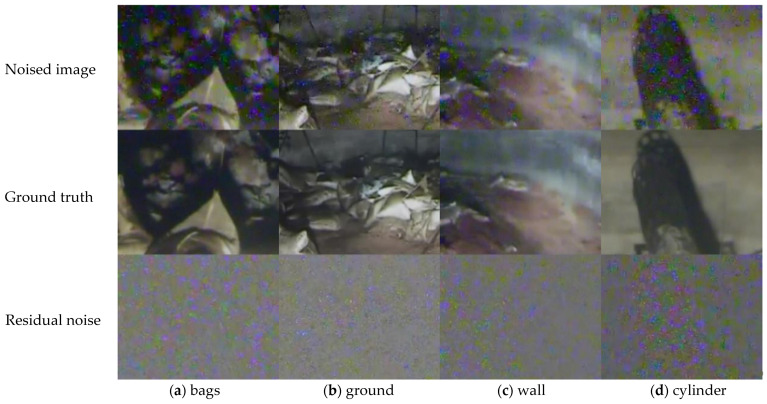
Four scenes of the nuclear radiation dataset. (**a**–**d**) Show different radiation scenes: bags, ground, wall, and cylinder. The first row shows the original noised images, the second row shows the clean ground truth images, and the third row shows the residual noises.

Our method aimed at removing strong noises in radiation scenes based on CNN, so we made a nuclear radiation dataset composed of noise-and-clean image pairs. The original noised images sized 640 × 480 in this dataset are all captured from the cameras on a special robot in a nuclear emergency accident. There are 37 scenes in the datasets, and each scene has 150 noised images and 1 clean image. Note that each clean image is obtained by averaging the 150 noised images. Finally, 2960 image pairs are used for training, 370 image pairs are used for validation, and 370 image pairs are used for testing, following the commonly used dataset splitting ratio 8:1:1. Figure 7 shows four challenging scenes of our nuclear radiation dataset.

As shown in Figure 7, the residual noise maps in the third row have no obvious regulations in term of color, shape, and distribution, while the clean images in the second row have much better perceptual satisfaction than the original noised images in the first row, indicating that the nuclear radiation dataset is applicable.

### 4.2. Public Synthetic Noise Datasets

In order to verify the generalization ability of our network, we carried out experiments on public synthetic noise datasets for three synthetic noise removal tasks, including gaussian noise, text noise, and impulse noise.

The training set is the widely used Pristine image dataset [36], which contains 3859 color photographs. The validation set and the testing set are the widely used McMaster [13] dataset and the Kodak [37] dataset, which contain 18 and 24 high quality images, respectively.

The synthetic noises are added online, and they have the same mean value of 0. The noise variances in the specific denoising experiments are 25, 50, 75.

### 4.3. Training and Testing Details

We use the same training and testing strategies in all denoising experiments to ensure fairness:

- The training sets are all image patches cropped from training image pairs with windows size 50 × 50 and stride 40, while the validation set and the testing sets are the images pairs with their original sizes.
- In training stages, the default batch sizes are 128, and the images patches *X* are normalized by *X*/255 typed Float32. In addition, the optimization methods are those adopted in Adam [38] with the initial learning rate 0.001, and the network initialization methods are those adopted in Kaiming [2]. Validations are performed and recorded at the end of each epoch.
- In testing stages, the batch sizes are 1, and the inference platform is TITAN XP. Note that all networks are trained for 50 epochs, and we choose the models with the highest PSNR on the validation set for testing.

### 4.4. Evaluation Metrics

We adopted three commonly used evaluation metrics for the image restoration tasks, including peak signal-to-noise ratio (PSNR), structural similarity (SSIM) [39], and mean opinion scores (MOS) [28].

The unit of PSNR is decibels (dB), and its calculation formula is:

(5) $$\left\{\begin{array}{l} MSE(x,y)=\frac{1}{mn}\sum_{i=0}^{m-1}\sum_{j=0}^{n-1}{\left[y(i,j)-y(i,j)\right]}^{2}\\ \mathrm{PSNR}(x,y)=20\log_{10}(\frac{\mathrm{MAX}}{\sqrt{\mathrm{MSE}(x,y)}}) \end{array}\right.$$

where *x* and *y* are the input images; *MAX* is the maximum value of the images’ grayscale; *Y* and *Y*_1_ are the noised image and the denoised result, respectively. Since the input data of the network *X* has been normalized by *X*/255, we set *MAX* = 1. It can be seen from the formula that the larger the PSNR value, the smaller the mean square error (MSE) between the noised image and the denoised result, i.e., the less the distortion of the reconstructed image.

The mean pixel value of an image denotes the estimate of brightness, while the standard deviation denotes the estimate of contrast, and covariance is a measure of structural similarity. Therefore, the SSIM metric combines the information from the three estimators and comprehensively evaluates the effect of image restoration. For the two given images, their structural similarity SSIM is defined as:

(6) $$\mathrm{SSIM}(x,y)=\frac{\left(2\mu_{x}\mu_{y}+c_{1})(2\sigma_{xy}+c2\right)}{\left({\mu_{x}}^{2}+{\mu_{y}}^{2}+c_{1})({\sigma_{x}}^{2}+{\sigma_{y}}^{2}+c_{2}\right)}$$

where *x* and *y* are the input images; *μ_x_*, *σ_x_*^2^ are the mean and variance of *x*, respectively; *μ_y_*, *σ_y_*^2^ are the mean and variance of *y*, respectively; *σ_xy_* is the covariance between *x* and *y*; *c*_1_ and *c*_2_ are two constants that maintain the stability of the calculation, and we set *c*_1_ = 0.01^2^, *c*_1_ = 0.03^2^. Unlike PSNR that calculates the entire image, SSIM calculates the image patches with a sliding window sized *m* × *m* (we set *m* = 11). The ultimate global SSIM value is the mean of local SSIM values from all patches. SSIM is also a good quantitative metric to represent the quality of image restoration.

In addition, we performed mean opinion scores (MOS) to quantitatively quantify the perceptual satisfaction of the restored image. Specifically, we asked 20 volunteers with different ages and occupations, and required them to score the images (ranged 0 to 5). The ultimate MOS of the image is the mean value from all the volunteers.

## 5. Results

In this session, the proposed method is compared with other denoising methods on our nuclear radiation dataset with respect to quantitative and qualitative evaluations. We performed experiments on two versions of our method: the normal version with 32 channels in its backbone, and the tiny version with 16 channels in its backbone.

### 5.1. Quantitative Comparisons

Table 1 lists the averaged Frames per Second (FPS) and quantitative metrics on our nuclear radiation dataset compared with six traditional image prior-based methods and six latest CNN-based methods. The traditional image prior-based methods are tested on a CPU (Intel Core i7-6700), and the Floating Point of Operations (FLOPs) [40] of CNN-based methods are based on the same input tensor shape (1, 3, 480, 640).

The existing denoising methods for nuclear radiation noise removal are shown in rows (1)–(3) in Table 1. Though the methods achieve real-time performance (more than 12 FPS), their quantitative metrics are not outstanding. The general image prior-based methods in rows (4)–(6) perform better than the methods in row (1)–(3), but they are far from the real-time FPS. The CNN-based methods in rows (7)–(8) have high PSNR and SSIM, but their FPS are less than 12. In this case, our method achieves the highest PSNR (33.81) and the highest SSIM (0.934), while our model still has a real-time FPS (14.57).

It is worth noticing that our tiny version achieves the lowest FLOPs (23.14 G), the fewest model parameters (75.25), and the highest FPS (18.38). Meanwhile, the PSNR and SSIM metrics of our tiny version are still comparable with other CNN-based methods. The reason is: although there are fewer parameters, the structure of our network is more complex, which ensures its feature learning ability.

Therefore, we can use the complexity of the network structure in exchange for the miniaturization of the model, and this strategy achieves better results.

### 5.2. Qualitative Comparison

**Figure 8:**
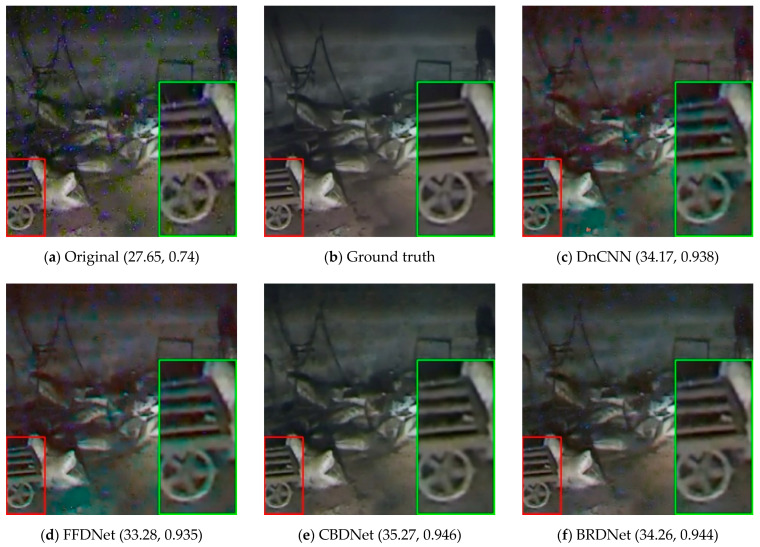

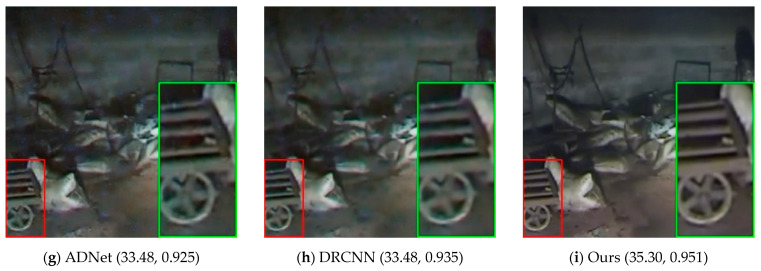
Comparison of results of a nuclear radiation image. (**a**) is the original noised image; (**c**–**h**) are the denoised results obtained from six popular CNN-based methods; (**i**) is our denoised result. There is a value tuple under each subfigure, and the values in the tuple are, respectively, the peak signal-to-noise ratio (PSNR) and structural similarity (SSIM) calculated with the ground truth clean image shown in (**b**).

We compare our intuitive results with those obtained from the popular CNN-based methods in nuclear radiation scenes, and locally analyze their texture retention. Figure 8 shows the denoised results of a nuclear radiation scene. The red box area is the cart with rich texture, and the green box area is the area enlarged twice.

In Figure 8, it can be seen from the top-left noised image that the nuclear noises have irregular aggregated shapes and different colors (blue and green), and there are burrs on the edge of the cart in the image. Although all the CNN-based denoising methods can remove the noises well, they still have some dissatisfied problems. Specifically, DnCNN [16], FFDNet [17], ADNET [20] and DRCNN [21] give the restored image different degrees of color distortion (e.g., some regions are prone to being green). BRDNet [19] and CBDNet [18] do well in color fidelity, but the textures of the wheel in the image are a little fuzzy. However, the result of our method has no color distortion while its edges are clear, indicating that our method does have advantages in terms of texture retention.

**Figure 9:**
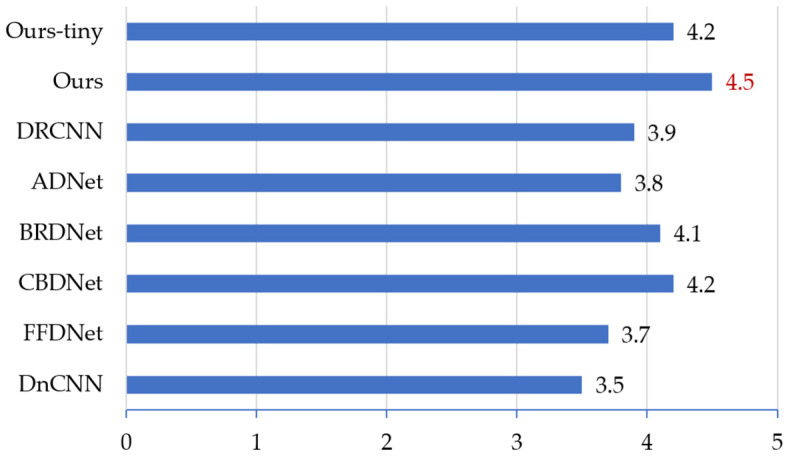
Mean opinion scores (MOS) comparison of denoising results on the nuclear radiation dataset.

All the denoised results of our dataset (with 37 scenes) from the above methods were shown to volunteers, obtaining the MOS metrics of the denoising methods. Figure 9 shows the results.

As shown in Figure 9, the result of our method is the same as the results of DRCNN and CBDNet that in the scores are more than four. However, our MOS is the highest (4.5), while our model is much more lightweight, so the overall performance of our method is the best.

## 6. Discussion

In this session, we carried out more detailed discussions about our network. First, a series of ablation experiments was performed to find the best hyperparameters and verify the superiority of the components. Then, we evaluate other performances of our network, including the trainability and the generalization ability.

### 6.1. Ablation Experiments for Hyperparameters

#### 6.1.1. Choice of Loss Function

**Figure 10:**
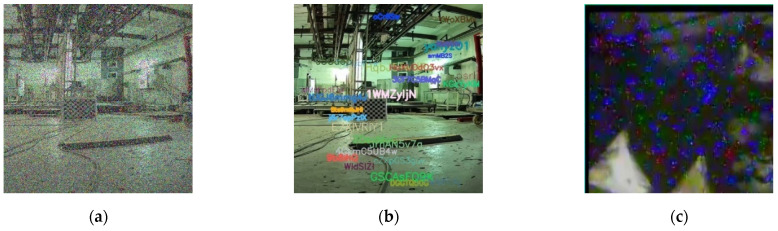
Visualization of different noise types. (**a**,**b**) Are synthetic impulse noise and text noise with the variance of 50 and 25, respectively; (**c**) is the real noise in our nuclear radiation dataset.

As mentioned in Section 3.3, the selection of loss functions is related to the noise type of the denoising task. We visualized the two synthetic noise types and compared them with our nuclear radiation noise. Figure 10 shows the visualizations.

**Figure 11:**
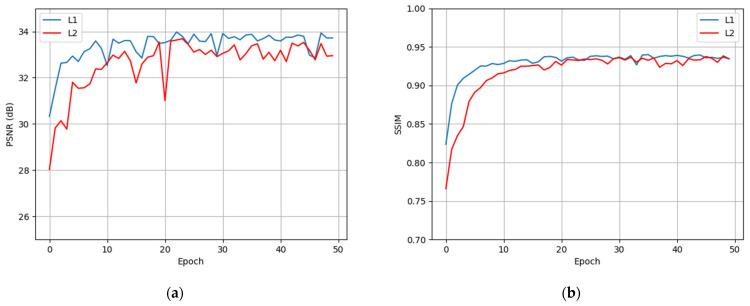
Quantitative metrics of different loss functions in the training stages. (**a**,**b**) Respectively, show the change of PSNR and SSIM with the increase in epoch. The blue curves in (**a**,**b**) denote that the model is optimized by *l*_1_ loss, and the red curves are for *l*_2_ loss.

It can be seen from Figure 10c that our nuclear radiation scene image is much different from the impulse noise in Figure 10a and the text noise in Figure 10b. However, the nuclear radiation noise is a bit similar to the combination of the two synthetic noises. On the one hand, the independent noise points in the nuclear radiation images are similar to the impulse noise shown in Figure 10a; on the other hand, the noise blocks in the nuclear radiation images are similar to the text noise shown in Figure 10b, as they are all aggregated with irregular shapes and colors. Therefore, we believe that *l*_1_ loss is more conducive to optimization than *l*_2_ loss in the task of nuclear radiation noise removal due to the same properties in the tasks of impulse noise removal and text noise removal [10]. To prove this point, we carried out ablation experiments between *l*_1_ and *l*_2_ losses. Respectively, the formulas of the two losses are:

(7) $$\left\{\begin{array}{l} l_{1}(x,y)=\;|\;x-y\;|\\ l_{2}(x,y)={\left(x-y\right)}^{2} \end{array}\right.$$

where *x* is the noised input and *y* is the denoised output. Their performances in the training stages are shown in Figure 11.

As shown in Figure 11, the metrics of *l*_1_ loss are almost above that of *l*_2_ loss, indicating that *l*_1_ loss is easier to optimize. It is worth noticing that *l*_1_ loss converges faster as shown in epochs 0–10, and its training curve is more stable (as shown in epoch 20 that the metric of *l*_2_ loss has an obvious jitter). This result demonstrates that the nuclear radiation noise is similar to the combination of impulse noise and text noise, and our choice to use *l*_1_ loss instead of the commonly used *l*_2_ loss is correct.

#### 6.1.2. Tricks of the NLU

The proposed noise learning unit NLU is composed of three important parts: Feature Extractors (FE), Receptive Field Blocks (RFB), and Attention Blocks (AB). FE contain two different backbones: Multi-kernel Modules (MKM) and Residual Modules (RM). The whole network adopted Mish activation function to enhance the feature learning ability and used asymmetric convolutions to reduce the number of parameters.

For the feature extractors (MKM and RM), Table 2 shows the comparisons in terms of PSNR, SSIM, and FLOPs. In this experiment, MKM and RM adopted Mish activation and asymmetric convolutions, while the structure of vanilla layers is the same as the structure of DnCNN [16], i.e., the input and output are connected by a subtractive skip connection, and each convolution layer is followed by a batch normalization BN and an activation function ReLU. Note that no dilated convolutions and asymmetric convolutions in vanilla layers, and their convolutional kernel sizes are all 3 × 3. In addition, the FLOPs are calculated with the input tensor shape (1, 3, 480, 640).

It can be seen from Table 2 that the PSNR and SSIM metrics of the MKM or RM are all close to that of vanilla layers, but the FLOPs of our model are much fewer than those of vanilla layers due to the fewer channels and the asymmetric convolutions. It is worth noticing that, when we parallelly combined the MKM and RM, the PSNR and SSIM metrics became much higher than that of the previous methods. This huge gain shows that we can learn more image information from our special feature extractor. Though the convolution layers become twice those of vanilla layers, the model is more lightweight.

In order to verify the effectiveness of other techniques, i.e., RFB, AB, and Mish, we conducted ablation experiments through a grid search. Table 3 shows the results of the experiments.

In Table 3, FE represents our feature extractor, i.e., the parallelly combined MKM and RM. If the Mish activation function is not selected, we use ReLU instead. It can be seen from experiments (1)–(4) of the table that RFB, AB, and Mish can individually bring certain improvements for PSNR and SSIM, indicating that the three extra techniques are effective. Although the pairwise combination between RFB and other tricks in experiments (5)–(7) has a decrease in PSNR compared with experiments (1)–(4), its SSIM metric has been improved slightly. More importantly, it can be seen from the last experiment that the improvement from using all the three techniques together is huge, especially for the PSNR that improved by 0.42 compared to the FE-only in experiment (1).

#### 6.1.3. Effectiveness of the TLU

**Figure 12:**
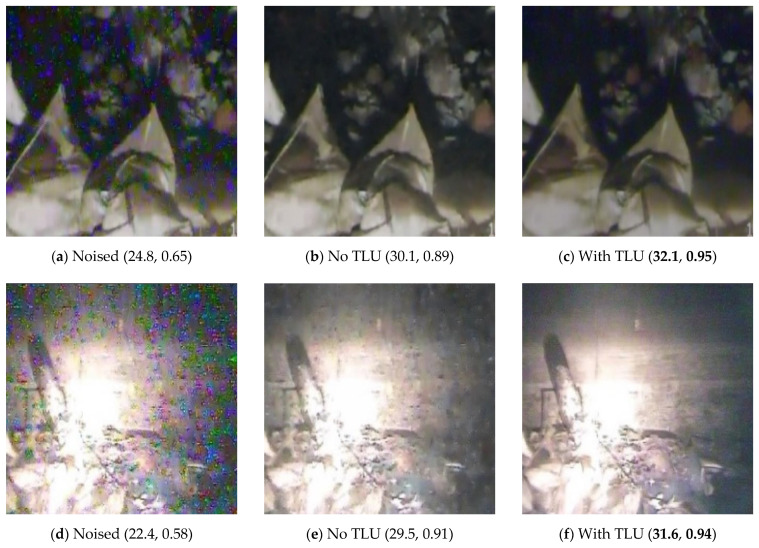

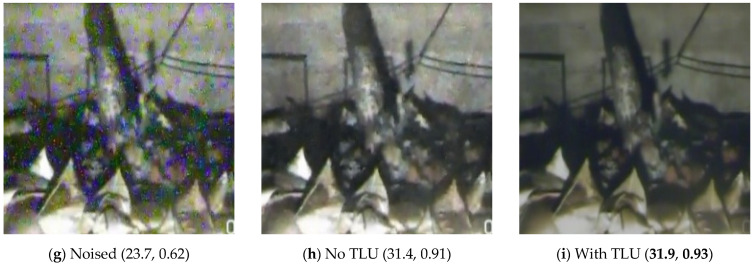
Denoising results of our network with and without the TLU. The first column containing (**a**,**d**,**g**) is the original noised images; the second column containing (**b**,**e**,**h**) is the denoised results of the model without the TLU; the third column containing (**c**,**f**,**i**) is the denoised results of the model with the TLU. There is a value tuple under each subfigure. The first value in the tuple is the PSNR calculated with the ground truth clean image, and the second number in the tuple is the SSIM. For each scene, the best quantitative results are marked in boldface.

Unlike other popular CNN-based denoising methods such as [16,17,18,19,20], we considered the learning of textures through adding a lightweight TLU at the bottom of the NLU, so as to make the final denoising results more perceptually satisfactory. Note that our method is different from the noise learning part and the texture learning part in the previous work [21] in that the TLU is identical to the NLU, and the two parts share the same loss function MSE to optimize. We use *l*_1_ loss to optimize the NLU and use *l*_2_ loss to optimize a tiny TLU composed of three convolution layers. Figure 12 shows the results in 3 challenging nuclear radiation scenes.

As shown in Figure 12, the results with the TLU have better perceptual effects than the results without the NLU in all scenes, i.e., the dark scene, bright scene, and regular scene. Meanwhile, for the quantitative metrics, the results with the TLU are also higher in terms of PSNR and SSIM than the results without the TLU. Therefore, the TLU can obviously improve the overall performance of our image restoration network.

### 6.2. Evaluations of Other Performance

#### Trainability

As conducted in Section 5.1, our network achieves the balance of denoising effect and model complexity, especially for our tiny version that achieves the best real-time performance. In this case, we evaluate the trainability of our model compared with six latest CNN-based denoising methods. Specifically, we test the denoising models with the same training times (6 h), and the occupied GPU memories are recorded, as shown in Table 4.

As shown in rows (1)–(7) in Table 4, with the same batch sizes and training times, our model obtained comparable PSNR and SSIM with other big models. The reason is that our tiny model optimizes more iterations in the same training times. More importantly, as shown in row (8), when we double the batch size, the PSNR and SSIM of our model become much higher than those of other models with the same training times. The reason is that the doubled batch size improves the utilization rate of GPU, and the model has more iterations for optimization in the same training time. Therefore, our method has better trainability compared with other popular CNN-based denoising methods, benefiting from its much smaller model size.

### 6.3. Generalization Ability

To examine the generalization ability of our network, we performed experiments on the Kodak dataset for denoising synthetic noises compared with five fast CNN-based methods. Table 5 and Table 6 show the comparison of the PSNR and SSIM metrics, respectively.

As shown in Table 5 and Table 6, our method achieves the highest PSNR and SSIM compared with other popular CNN-based denoising methods, while our tiny version also has comparable results though its model size is the smallest. The results indicates that our network not only performs well in nuclear radiation scenes, but also has good generalization ability in terms of other denoising tasks.

## 7. Conclusions

In order to remove the complex and strong-level noise in radiation scene images, we designed a lightweight network composed of a noise learning unit (NLU) and a texture learning unit (TLU). The proposed network applied multi-scale kernel convolution, receptive field blocks, Mish activation, and asymmetric convolution to denoising tasks for the first time, and these tricks provided substantive improvements.

Compared with 12 denoising methods including 6 traditional image prior-based methods and 6 latest CNN-based methods on our nuclear radiation dataset, the proposed method has the real-time FPS with the highest PSNR and SSIM metrics.

In addition, compared with the six CNN-based methods, our network had the highest MOS score and the best perceptual effects on our nuclear radiation dataset, and obtained the highest quantitative metrics on the public Kodak dataset for removing synthetic Gaussian noise, text noise, and impulse noise.

Therefore, the strategy of using the complexity of the network structure in exchange for the miniaturization of the model is effective, and the proposed method commendably solves the problem of strong noise removal in nuclear radiation scenes.

## Figures and Tables

**Table 1:** Quantitative results of denoising methods on nuclear radiation dataset. In each column, compared with all methods, the best value is marked in boldface.

Row	Method	FLOPs (G)	Parameters (K)	FPS	PSNR	SSIM
1	Wang [6]	-	-	12.29	29.21	0.875
2	Zhang [7]	-	-	14.43	30.14	0.880
3	Yang [8]	-	-	13.17	30.33	0.847
4	NLM [13]	-	-	0.0098	30.85	0.889
5	BM3D [11]	-	-	0.0058	31.52	0.895
6	WNNM [15]	-	-	0.0012	31.24	0.899
7	BRDNet [19]	343.92	1120	8.06	32.79	0.922
8	CBDNet [18]	189.3	4370	4.97	33.11	0.927
9	DnCNN [16]	171.86	558.4	14.57	32.87	0.927
10	FFDNet [17]	65.72	854.69	14.43	32.03	0.915
11	ADNet [20]	160.5	521.49	12.53	32.12	0.919
12	DRCNN [21]	343.71	1120	12.33	33.01	0.922
13	Ours	68.15	221.76	14.57	**33.81**	**0.934**
14	Our-tiny	**23.14**	**75.25**	**18.38**	32.51	0.923

**Table 2:** Denoising effects of different feature extraction networks. The best quantitative results are marked in boldface.

Backbone	Channels	Layers	FLOPs (G)	PSNR	SSIM
Vanilla	64	15	171.84	33.35	0.935
MKM only	32	15	30.66	33.33	0.938
RM only	32	15	31.92	33.37	0.936
MKM–RM	32	30	62.04	**34.56**	**0.951**

**Table 3:** Grid search results for multiple techniques. The best quantitative results are marked in boldface.

Experiment	FE	RFB	AB	Mish	PSNR	SSIM
1	✓				33.56	0.938
2	✓	✓			33.58	0.941
3	✓		✓		33.64	0.939
4	✓			✓	33.75	0.940
5	✓	✓	✓		33.29	0.942
6	✓	✓		✓	33.07	0.941
7	✓		✓	✓	33.62	0.940
8	✓	✓	✓	✓	**33.98**	**0.944**

**Table 4:** Training and testing details of different denoising methods on the nuclear radiation dataset. The best quantitative results are marked in boldface.

Row	Methods	Training Time (hour)	Batch Size	Occupied GPU Memory (M)	PSNR	SSIM
1	DnCNN [16]	6	128	5818	30.74	0.861
2	FFDNet [17]	6	128	5214	30.93	0.865
3	CBDNet [18]	6	128	6412	31.10	0.879
4	ADNet [20]	6	128	4172	31.02	0.874
5	BRDNet [19]	6	128	6374	31.45	0.877
6	DRCNN [21]	6	128	6512	30.97	0.880
7	Our-tiny	6	128	2906	31.07	0.883
8	Our-tiny	6	256	5214	**31.79**	**0.894**

**Table 5:** Comparison of PSNR metrics of 6 latest CNN -based methods on the Kodak dataset. For each noise type, the highest PSNRs are marked in boldface.

	Noise Types	Gaussian	Text	Impulse
	Noise Levels	25/50/75	25/50/75	25/50/75
1	DnCNN	31.93/29.98/25.46	29.33/28.80/25.77	36.70/32.93/28.58
2	FFDNet	31.01/29.56/25.62	30.40/29.01/26.58	36.62/34.68/28.96
3	CBDNet	32.12/29.96/25.74	31.61/29.25/26.96	38.75/35.08/29.92
4	ADNet	31.11/28.66/25.62	30.43/28.01/25.57	36.62/34.68/28.66
5	BRDNet	32.23/30.52/25.77	31.18/29.35/26.17	38.97/35.71/29.94
6	DRCNN	31.11/29.66/25.62	30.40/28.01/26.57	36.62/34.68/28.81
7	Ours-tiny	32.31/30.44/25.71	31.57/29.01/27.01	39.04/35.51/29.82
8	Ours	**32.41/30.54/25.78**	**32.74/29.86/27.36**	**41.21/35.74/30.86**

**Table 6:** Comparison of SSIM metrics of 6 latest CNN-based methods on the Kodak dataset. For each noise type, the highest SSIMs are marked in boldface.

	Noise Types	Gaussian	Text	Impulse
	Noise Levels	25/50/75	25/50/75	25/50/75
1	DnCNN	0.925/0.859/0.666	0.901/0.801/0.703	0.922/0.924/0.808
2	FFDNet	0.929/0.860/0.672	0.902/0.792/0.717	0.932/0.938/0.806
3	CBDNet	0.938/0.862/0.702	0.920/0.789/0.737	0.972/0.959/0.880
4	ADNet	0.929/0.856/0.701	0.914/0.765/0.672	0.937/0.935/0.836
5	BRDNet	0.932/0.855/0.703	0.926/0.828/0.729	0.913/0.951/0.854
6	DRCNN	0.938/0.854/0.701	0.922/0.811/0.737	0.962/0.953/0.826
7	Ours-tiny	0.938/0.860/0.704	0.921/0.833/0.749	0.978/0.955/0.879
8	Ours	**0.940/0.866/0.713**	**0.936/0.838/0.755**	**0.983/0.960/0.882**

## Data Availability

Not applicable.
